# Variation of FMRP Expression in Peripheral Blood Mononuclear Cells from Individuals with Fragile X Syndrome

**DOI:** 10.3390/genes15030356

**Published:** 2024-03-13

**Authors:** Jamie L. Randol, Kyoungmi Kim, Matthew D. Ponzini, Flora Tassone, Alexandria K. Falcon, Randi J. Hagerman, Paul J. Hagerman

**Affiliations:** 1 Department of Biochemistry and Molecular Medicine, School of Medicine, University of California, Davis, Davis, CA 95616, USA; 2 Medical Investigation of Neurodevelopmental Disorders (MIND) Institute, UC Davis Health, Sacramento, CA 95817, USA; 3 Department of Public Health Sciences, School of Medicine, University of California, Davis, Davis, CA 95616, USA; 4 Department of Pediatrics, School of Medicine, University of California, Davis, Sacramento, CA 95817, USA

**Keywords:** *FMR1*, fragile X syndrome, autism, full mutation, TR-FRET, mosaicism, intellectual disability

## Abstract

Fragile X syndrome (FXS) is the most common heritable cause of intellectual disability and autism spectrum disorder. The syndrome is often caused by greatly reduced or absent protein expression from the *fragile X messenger ribonucleoprotein 1* (*FMR1*) gene due to expansion of a 5′-non-coding trinucleotide (CGG) element beyond 200 repeats (full mutation). To better understand the complex relationships among *FMR1* allelotype, methylation status, mRNA expression, and *FMR1* protein (FMRP) levels, FMRP was quantified in peripheral blood mononuclear cells for a large cohort of FXS (*n* = 154) and control (*n* = 139) individuals using time-resolved fluorescence resonance energy transfer. Considerable size and methylation mosaicism were observed among individuals with FXS, with FMRP detected only in the presence of such mosaicism. No sample with a minimum allele size greater than 273 CGG repeats had significant levels of FMRP. Additionally, an association was observed between *FMR1* mRNA and FMRP levels in FXS samples, predominantly driven by those with the lowest FMRP values. This study underscores the complexity of *FMR1* allelotypes and FMRP expression and prompts a reevaluation of FXS therapies aimed at reactivating large full mutation alleles that are likely not capable of producing sufficient FMRP to improve cognitive function.

## 1. Introduction

Fragile X syndrome (FXS) is an X-linked neurodevelopmental disorder caused by reduced or absent expression of the protein product (FMRP) of the fragile X messenger ribonucleoprotein 1 (*FMR1*) gene. In the vast majority of FXS cases, expansion of a CGG trinucleotide, located in the 5′ untranslated region of *FMR1,* to above 200 repeats (full mutation, FM) triggers hypermethylation and silencing of the gene, with consequent reduction/absence of the gene product [1,2,3]. Such expansion mutations constitute the most common heritable cause of intellectual impairment and of autism spectrum disorder [4,5,6,7,8,9]. FMRP is an RNA-binding protein with a plethora of binding partners and functions, both in the CNS and in peripheral tissues. Within the CNS, FMRP regulates synaptic plasticity, neural development, and cognitive function, primarily by regulating the translation, transport, and stability of many mRNAs [10]. The loss of FMRP results in intellectual disability and, often, attention-deficit/hyperactivity disorder (ADHD), anxiety, autism, and other neurological and behavioral symptoms [11,12,13]. Predictably, decreasing FMRP levels are associated with increasing severity of FXS [14], lowered IQ [15], and other neurological disorders including bipolar disorder, depression, and schizophrenia [16,17,18].

Increases in *FMR1* CGG-repeat length beyond the normal range (<55 CGG repeats) are known to cause various fragile X-associated conditions. CGG alleles with repeats between 55 and 200 are termed “premutation” (PM), given their propensity to expand to FM alleles within one generation. Unlike FM alleles, PM alleles are generally unmethylated and produce excess mRNA and relatively normal to slightly reduced levels of FMRP [19]. Therefore, individuals with PM alleles usually exhibit normal cognitive function. However, they are predisposed to develop other physical and psychiatric disorders. Chief among them is the neurodegenerative disorder fragile X-associated tremor/ataxia syndrome (FXTAS), which can cause intention tremor, cerebellar ataxia, and cognitive decline, similar to symptoms seen in Parkinson disease and Alzheimer disease [19].

Often, individuals with FXS exhibit allelic mosaicism, possessing multiple alleles that can distribute across FM–PM or PM–normal size boundaries. It is not uncommon for an FXS individual to have unmethylated normal or PM alleles in addition to multiple FM alleles [19]. Moreover, methylation mosaicism is also a frequent occurrence among those with FM alleles. The large degree of mosaicism contributes to varying levels of FMRP production and is a confounding factor in determining the relationship between CGG repeat size, methylation, transcription, and translation at the *FMR1* locus, particularly in the upper PM and FM range.

Accurately measuring low FMRP levels in FXS is important for better understanding the relationship between CGG-repeat size and methylation class and its effect on the development of potential treatments for fragile X-associated disorders. Various methods have been developed to measure FMRP. Some of the first methods, such as Western blotting, immunocytochemistry, or immunohistochemistry, were low-throughput, semiquantitative, and/or labor-intensive [20]. The introduction of assays using two unique antibodies to simultaneously bind FMRP has dramatically improved sensitivity and specificity [20,21,22,23]. In a previous study, we used one of these methods, time-resolved fluorescence resonance energy transfer (TR-FRET), to quantify the relationship between IQ and FMRP levels [15]. One advantage of TR-FRET is that it can occur in homogenous cell lysate, eliminating the need for multiple washing or separation steps.

The current study extends the use of homogeneous TR-FRET to explore the relationship between FMRP production and the complex size- and methylation-mosaic allelotypes found in FXS individuals using a large sample size (293 individuals with and without FXS) in an accessible cell type–peripheral blood mononuclear cells (PBMCs). A high degree of size and methylation mosaicism is observed for the FXS cohort, with significant levels of FMRP being produced only from size and/or methylation mosaics with alleles below or just above the PM–FM size boundary. Additionally, a reduction in translation efficiency was observed as the repeat size of the smallest allele increased in the FM range. Despite evidence of excess mRNA production in the unmethylated FM range, no PBMCs with their smallest allele above ~270 CGGs produced significant levels of FMRP.

## 2. Materials and Methods

### 2.1. Participants and Samples

Blood samples from males with FXS and male controls were used in this study. Males with FXS were diagnosed clinically after assessing the behavioral, cognitive, and physical phenotypes at the Fragile X Treatment and Research Center at the UC Davis MIND Institute at UC Davis Health. Females were excluded from this study, eliminating the confounding, variable contribution to FMRP levels from the unaffected X chromosome due to a broad range of activation ratios. Blood was collected at the MIND Institute, under protocols approved by the Institutional Review Board at the University of California, Davis. Between 2010 and 2017, blood was drawn from a total of 293 individuals (322 samples, including biological replicates): 155 individuals (170 samples, including biological replicates) diagnosed with FXS and 138 typically developing controls (152 samples, including biological replicates). From these individuals, 390 samples were processed, which included both technical replicates (same blood draw) and biological replicates (same individual, different date of blood draw). See Table 1 for descriptive statistics of all samples (technical replicates were represented only once) by allele class, CGG repeat size, and age. See Supplementary Information (Section S1) for methods of sample elimination.

### 2.2. PBMC Collection and Storage

PBMCs were collected by venipuncture in BD Vacutainer CPT tubes (BD, Franklin Lakes, NJ, USA) and processed following the manufacturer’s protocol to obtain mostly (~70–90%) lymphocytes. Cells were resuspended in RPMI 1640 (Gibco, Grand Island, NY, USA) with 10% dimethyl sulfoxide and partitioned into one to three aliquots for cryopreservation in liquid nitrogen until needed.

### 2.3. CGG Genotyping and Methylation Status

Genomic DNA was isolated from PBMCs using standard procedures (Qiagen, Valencia, CA, USA). CGG repeat sizing was carried out by a combination of PCR and Southern blot analysis, as previously reported [26,27]. For Southern blot analysis, DNA was digested with *Eco*RI and *Nru*I, fixed on a nylon membrane and hybridized with the *FMR1* genomic probe StB12.3, and labeled with Dig-11-dUTP by PCR (PCR Dig Synthesis Kit; Roche Diagnostics) following the protocol as previously described [27]. PCR analysis was performed using *FMR1*-specific primers (AmplideX PCR/CE, Asuragen, Austin, TX, USA); amplicons were visualized by capillary electrophoresis and analyzed using Gene Mapper software (Applied Biosystems, Waltham, MA, USA) [26]. Methylation status, including the percentage of methylation (% of methylated alleles), was determined by densitometric analysis of Southern blotting images, as described in [28]. See Table S1 for the molecular data of each sample.

### 2.4. FMR1 mRNA Expression Levels

Total RNA was isolated from 2.5 mL of peripheral blood collected in PAXgene blood RNA tubes using the PAXgene Blood RNA Kit (Qiagen, Valencia, CA, USA) and quantified using the Agilent 2100 Bioanalyzer system (Agilent, Inc., Sana Clara, CA, USA). cDNA synthesis and determination of *FMR1* mRNA expression levels were performed using real-time PCR (qRT-PCR). Three reference genes were used: β-glucuronidase (*GUS*) (probe and primers as described in [29]), hydroxymethylbilane synthase (*HMBS*; TaqMan™ Assay Hs0060927, ThermoFisher Scientific, Waltham, MA, USA), and hypoxanthine-guanine phosphoribosyltransferase (*HGPRT*; TaqMan™ Assay Hs02800695, ThermoFisher Scientific, Waltham, MA, USA). Details are as described in [29]. Relative RNA was calculated by normalizing to the mean *FMR1* mRNA value of control samples in this study. See Table S1 for molecular data by sample.

### 2.5. TR-FRET Assay

To avoid protein degradation, 200 µL of Dulbecco’s phosphate-buffered saline (DPBS; Gibco, Grand Island, NY, USA) supplemented with Roche cOmpleteTM Ultra Protease Inhibitor Tablets (MilliporeSigma, Burlington, MA, USA) was added to approximately 1 mL of frozen PBMCs. Cells were then thawed in a 37 °C dry bath with intermittent gentle vortexing. 

Cells were pelleted by centrifugation at 1500× *g* for five minutes at 4 °C, washed with 100–200 µL DPBS with protease inhibitor, then lysed in 85 µL of 1× Cisbio Human FMRP lysis buffer (CisbioUS, Bedford, MA, USA) supplemented with cOmplete^TM^ protease inhibitor, 0.25 U/µL Benzonase (Millipore Sigma, Burlington, MA, USA), and 2 mM MgCl_2_. Pellets were disrupted by pipetting followed by rotation at room temperature for 2–3 h. Lysates were then spun at 16,000× *g* for 6 min to pellet any debris or unlysed cells. The resulting supernatants were used to perform total protein concentration analysis using Pierce^TM^ BCA Protein Assay (Thermo Fisher Scientific, Rockford, IL, USA). 

The TR-FRET method was used to quantify FMRP using the Cisbio Human FMRP assay (CisbioUS, Bedford, MA, USA) following the manufacturer’s protocol. Individual lysates were diluted to two protein concentrations differing by a factor of two, each within the range of 0.75 to 6.3 µg total protein in supplemented lysis buffer. Ten microliters of each total protein concentration were loaded in quadruplicate in a 384-well Opti-Plate (Perkin Elmer, Boston, MA, USA). Ten microliters of homogenous time-resolved fluorescence technology pre-mixed antibodies were added to each well. The FRET plate was rocked overnight for 18 h at room temperature and then read on the VictorX5 (PerkinElmer, Waltham, MA, USA). TR-FRET measurements occurred over a 400 µs window after a 50 µs delay to allow the decay of short-lived (ns) background fluorescence, such as from direct excitation of the acceptor. Readings at 615 nm (donor) and 665 nm (acceptor) were taken and ratios calculated as Ratio = (fluorescence at 665 nm/fluorescence at 615 nm) × 10^4^. The fractional change in this ratio was computed by ΔF% = (Ratio_sample_ − Ratio_lysis buffer_/Ratio_lysis buffer_) × 100 and used to determined relative FMRP concentrations (below).

### 2.6. FMRP Quantification

#### 2.6.1. Calculations

FMRP levels were quantified by interpolating ΔF% on a standard curve using a fibroblast fiducial line run alongside PBMC samples from study participants. The same fibroblast fiducial was used in the prior FRET analysis of Kim et al. [15]. Samples with ΔF% > 65 were interpolated using a four-factor fit generated from 0 to 3.5 µg total protein of the fiducial to account for the non-linearity of the model. However, for samples with ΔF% ≤ 65, interpolations used a linear fit generated for 0 to 0.4 µg of the fiducial. This allowed for negative ΔF% replicates to be interpolated and more accurate FMRP determination for samples with FMRP at or near zero. A ΔF% of 65 was chosen as the value to unite the two models as the interpolated FMRP values for both models were approximately equal at this ΔF% value. Next, interpolated FMRP values were corrected for PBMC total protein loaded (FMRP/µg). Finally, all corrected FMRP values were normalized to the mean corrected FMRP for individuals with control alleles and known mRNA levels (FMRP_rel_). See Table S1 for molecular data by sample. See Supplementary Information (Section S2) for method of extreme outlier removal.

#### 2.6.2. Significance of the Presence of FMRP

FMRP significance was determined by correcting raw FRET ratios to two types of negative controls. FRET ratios, rather than ΔF%, were used to determine FMRP significance, as these are the raw readings from the Victor X5 before further uncertainty is introduced by interpolation at the low end of the standard curve. First, wells containing only lysis buffer in the absence of total protein were used to determine background fluorescence on a plate-to-plate basis. Second, a PBMC sample (195-13) from a participant carrying a 300 Kb deletion encompassing the entire *FMR1* gene [25] was included in the assay to identify background fluorescence in the presence of total protein and the absence of FMRP. Sample FRET ratios were corrected first to the median FRET ratio for its plate’s lysis buffer control and then to the median lysis buffer-corrected FRET ratio of the deletion control. This process was carried out separately for the two protein concentrations of each sample. Significant presence of FMRP was then determined via one-sided, one-sample *t*-tests on the doubly corrected FRET ratios, testing the hypothesis that the mean FMRP concentration level is greater than zero. A sample was determined to have statistically significant FMRP (FMRP(+)) if the higher concentration had a significant corrected FRET ratio greater than 0 (i.e., *t*-test *p*-value < 0.05). That is, samples whose lower concentration was significant while the higher concentration was not significant were considered not significant for FMRP overall (FMRP(−)), as the assay is more variable for lower concentrations of total protein. See Table S1 for molecular data by sample.

### 2.7. Statistical Analyses

To assess the effects of *FMR1* mRNA, unmethylated CGG repeat alleles, methylated CGG repeat alleles, percentage of methylation, and/or age on FMRP, regression analyses were performed using nested linear mixed-effects models to incorporate nested data structures for FMRP (technical replicates nested within biological replicates). The models included *FMR1* mRNA, age, unmethylated CGG repeats, methylated CGG repeats, and/or fraction methylated as fixed effects, a random intercept for biological and technical replicates, and a random slope for age-measured FMRP. The median CGG repeat size of alleles—calculated using all CGG repeats, unmethylated and methylated CGG repeats separately, and the lower bound of smears plus one-quarter the range of the smears—was used in the regression analyses. The first quartile of the smear range was used to more heavily weight smaller alleles more likely to contribute to FMRP. We first performed a regression analysis to assess the effect of *FMR1* mRNA on FMRP, controlling for age. We then fitted the following sequential models: (1) Model 1—assessing the effects of two parameters (*FMR1* mRNA and unmethylated CGG repeats) on FMRP, controlling for age; (2) Model 2—assessing the effects of two parameters (*FMR1* mRNA and methylated CGG repeats) on FMRP, controlling for age and fraction of methylated CGG repeats; and (3) Model 3—assessing the effects of three parameters (*FMR1* mRNA, unmethylated CGG repeats, and methylated CGG repeats) on FMRP, controlling for age and fraction of methylated CGG repeats, as listed below. 

Model 1: FMRP = *FMR1* mRNA + Age + CGG_unmethylated_

Model 2: FMRP = *FMR1* mRNA + Age + CGG_methylated_ + Fraction Methylated

Model 3: FMRP = *FMR1* mRNA + Age + CGG_unmethylated_ + CGG_methylated_ + Fraction Methylated

Finally, likelihood ratio tests (LRTs) were performed to evaluate the difference between two nested models. The first compared Model 1 and Model 3 in order to test the significance of methylated CGG repeats and fraction of methylation on FMRP while accounting for *FMR1* mRNA, unmethylated CGG repeats, and age. The second compared Model 2 and Model 3 in order to test the significance of unmethylated CGG repeats on FMRP while accounting for *FMR1* mRNA, methylated CGG repeats, and age. All the analyses were conducted using open-source R software (version 4.2.1).

## 3. Results

### 3.1. PBMCs Express Lower Levels of FMRP Relative to Fibroblasts

A control dermal fibroblast line was used as a fiducial to generate a standard curve for relative FMRP levels, both for PBMC samples in this study and for dermal fibroblasts in [15]. Interpolation on this standard curve followed by correction for total protein loaded produced a mean of 0.26 FMRP/µg for PBMCs with control alleles. That is, control PBMCs produce approximately 4-fold less FMRP for the same total protein than the control fibroblast fiducial line. Subsequent analyses were performed on FMRP values normalized to the mean of control PBMCs (0.26 FMRP/µg was normalized to 1.0 as the control PBMC mean). 

### 3.2. FMRP Levels Are Independent of Year of Blood Draw and Age

**Figure 1:**
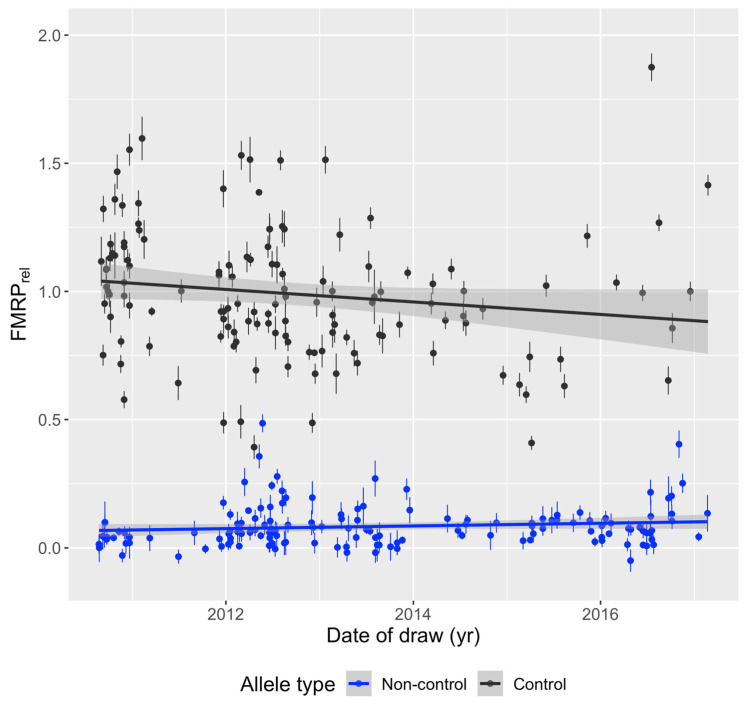
Relative FMRP levels by date of draw. Biological (same individual, different blood draw) and technical (same individual, same blood draw) replicates were removed at random to generate one sample per individual with known date of blood draw. Relative FMRP (mean ± SEM) from PBMCs was plotted against the date of blood draw and a linear regression was fitted, showing independence of FMRP on date of draw and thus length of time stored in liquid nitrogen. Control: *n* = 135, estimate/slope = −0.000067 relative FMRP units/day, *p*-value = 0.072. Non-control: *n* = 143, estimate/slope = 0.000014 relative FMRP units/day, *p*-value = 0.14.

**Figure 2:**
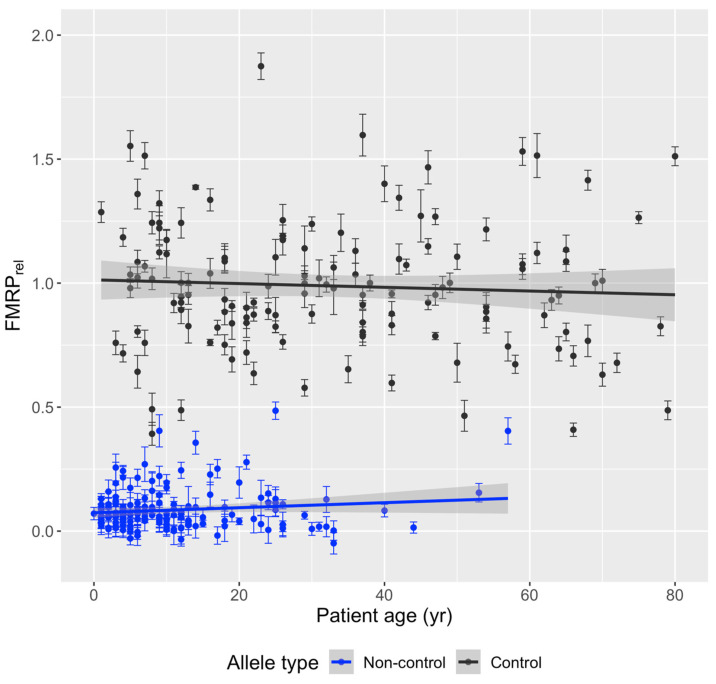
Relative FMRP levels by age at time of blood draw. Biological (same individual, different blood draw) and technical (same individual, same blood draw) replicates were removed at random to generate one sample per individual. Relative FMRP (mean ± SEM) from PBMCs was plotted against age of individual in years at the time of blood draw, and a linear regression was fitted, showing independence of FMRP on age. Control: *n* = 138, estimate/slope = −0.00075 FMRP units/day, *p*-value = 0.47. Non-control: *n* = 155, estimate/slope = 0.0010 FMRP units/day, *p*-value = 0.13.

One sample among biological and technical replicates per individual was randomly selected to generate a sample set with one sample per individual. Relative FMRP was plotted against the date of sample collection for research participants with known blood draw dates (Figure 1) to see if storage duration affected results. Linear regression analysis showed that FMRP levels are not significantly correlated with the date of the blood draw in individuals with control (*n* = 135 unique individuals with known blood draw dates, *p* = 0.07213) and non-control (*n* = 143 unique individuals with known blood draw dates, *p* = 0.143) alleles, suggesting that measurements of FMRP in cryopreserved whole PBMCs stored in liquid nitrogen were not sensitive to the number of years of sample storage (samples collected from January 2010 through March 2017) prior to assay of PBMC-isolated FMRP. Therefore, year of draw was not considered as a source of variation that would bias results in subsequent statistical analyses. Relative FMRP was also plotted by age at time of draw (Figure 2). Linear regression analysis showed no association between FMRP levels and age of individual at time of blood draw (control *p* = 0.4733, non-control *p* = 0.1277).

### 3.3. Individuals with FM Alleles Generally Have a Complex Genotype

**Figure 3:**
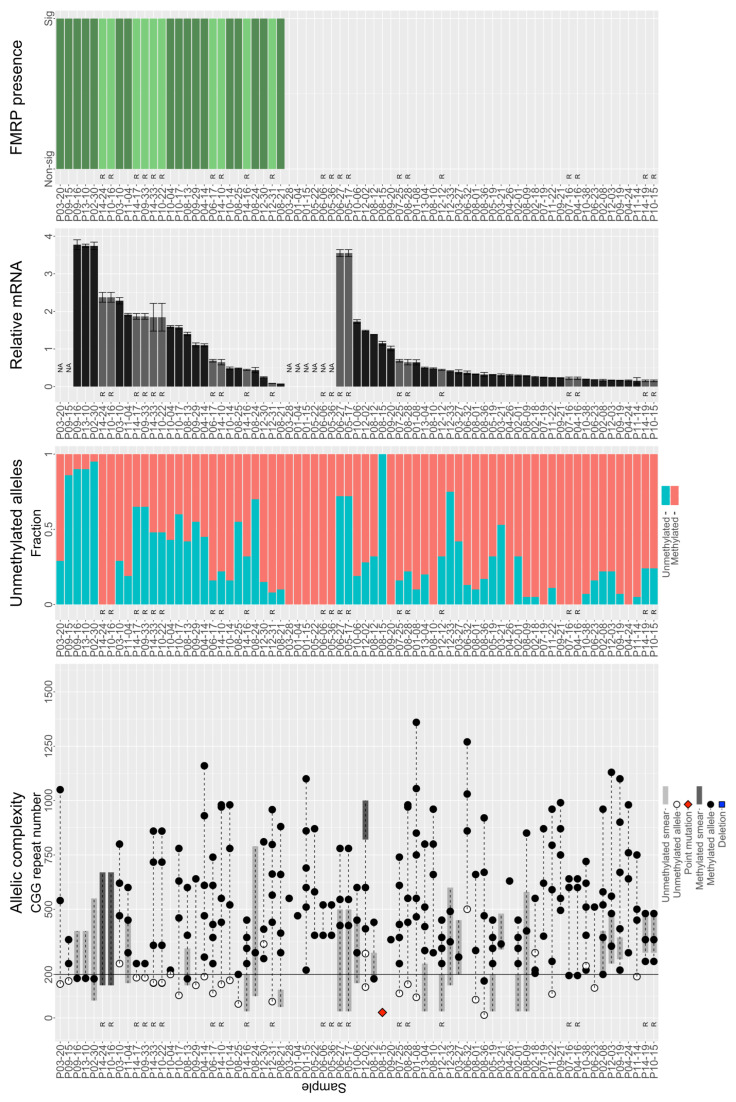

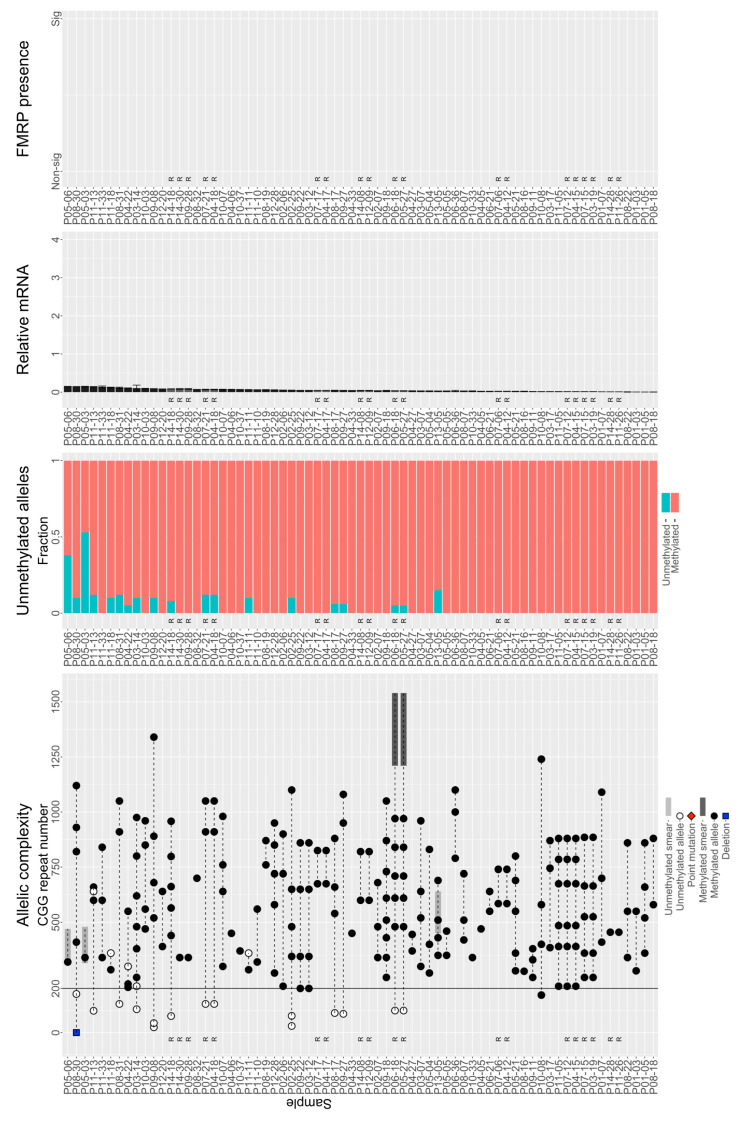

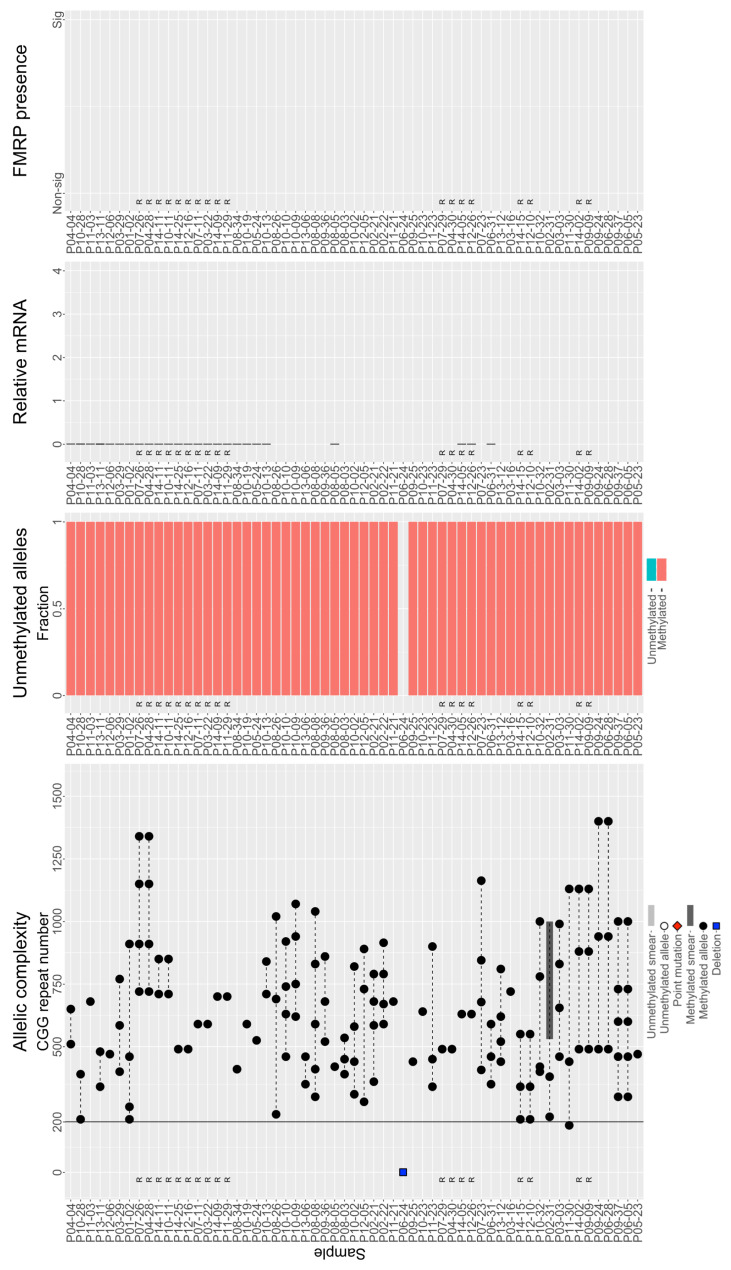
Genetic and molecular characteristics of non-control samples. All observations of each individual were included. Samples that had a technical replicate are denoted by the letter “R” and are shown in a lighter hue (light grey for mRNA level and light green for significant presence of FMRP). Samples were arranged first by FMRP significance then by mRNA level. Sample P08-15 contains a control allele with a point mutation. It produces mRNA, but its protein is not detected by the Cisbio FRET assay. Sample P06-24 is a deletion sample. *FMR1* is not present. Therefore, no methylation analysis was performed. *Allelic complexity*. All alleles identified via capillary electrophoresis are plotted by CGG repeat size and connected via a dashed line for each sample. Black circles: methylated alleles; white circles: unmethylated alleles; dark gray highlights: methylated smear range; light gray highlights: unmethylated smear range. *Unmethylated alleles*. Teal: fraction of unmethylated alleles; salmon: fraction of methylated alleles in a sample. *mRNA_rel_.* Relative mRNA (mean ± SE) was determined via RT-qPCR and normalized to the mean of samples with control alleles. Samples whose mRNA was not evaluated are denoted by “NA”. *FMRP status.* One-sample *t*-tests were performed on FRET ratios corrected to lysis buffer and deletion controls. A sample had significant FMRP when *p* < 0.05 for the highest concentration assayed. Only 27 samples with extended CGG repeats had significant levels of FMRP. All were mosaic for methylation and/or allele class.

Individuals with FM alleles generally are mosaic in terms of number, size, and/or methylation of alleles. That is, individuals with nominally FM alleles tend to have multiple alleles whose CGG repeats can span allele classes and be distinctly methylated (Table 1, Figure 3). Of 153 individuals with expanded CGG repeat alleles covering the FM range, only 26 (~17%) possessed a single detectable methylated allele; thus, 83% were mosaics of size (inter- or intra-class) and/or methylation status. Of these 127 individuals, there were 19 with methylation mosaicism, 1 who was mosaic for allele class, 41 who were mosaic for both methylation and allele class, and 66 with up to 8 discrete FM methylated alleles. Discrete allele sizes ranged from 13 to 1400 CGGs (Table 1).

Additional complexity was created by the presence of smears and degree of methylation. A smear is a quasi-continuous series of PCR products that differ in length by only a few CGG repeats, such that they appear as a smear rather than a discrete band when run on a gel or an electrophoretogram. Of the 153 individuals with nominally FM alleles, 20 had unmethylated smears and 4 had methylated smears. In 61 samples with unmethylated alleles, the fraction of unmethylated alleles ranged from 5% to 95% of all alleles present. Smear allele sizes ranged between 30 and 1540 CGGs (Table 1). Unsurprisingly, expanded-repeat samples with the largest significant FMRP levels were those with size and/or methylation mosaicism. Interestingly, not all samples with detectable RNA (those with larger FM CGG repeats) produced detectable FMRP (Figure 3).

### 3.4. Large Unmethylated and Methylated Alleles Produce mRNA

**Figure 4:**
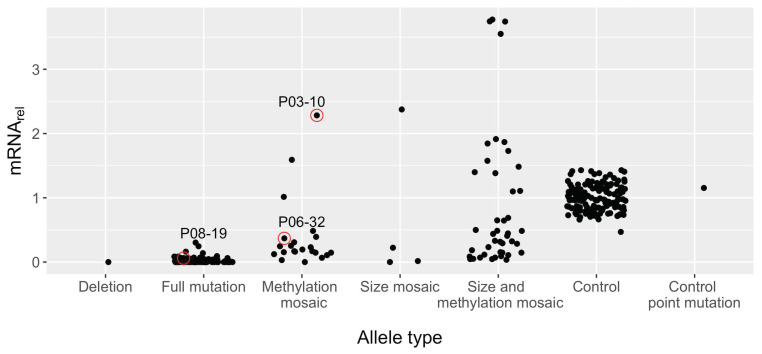
Distribution of relative *FMR1* mRNA within each allele type. Technical replicates were randomly removed to keep one observation per blood draw. Deletion: *FMR1* not present. Full mutation: only full-mutation methylated alleles. Methylation mosaics: some methylated and some unmethylated full-mutation alleles in the same individual. Size mosaic: some alleles under 200 CGG repeats and some full-mutation alleles. Size and methylation mosaics: some alleles smaller than 200 CGG repeats and some alleles of varying methylation status above 200 CGG repeats. Control: control alleles. Control point mutation: control allele with a point mutation that prevents detection by Cisbio FRET assay.

Methylation mosaics with CGG repeat alleles only in the FM range produced mRNA levels 0 to 2.28-fold that of the mean of control samples (Figure 4). Sample P03-10, with an unmethylated allele at 250 CGGs, produced the highest relative *FMR1* mRNA levels at 2.28 ± 0.085 (mean ± SEM), suggesting that smaller FM alleles can produce large quantities of mRNA, even above the range for samples with control alleles (0.47–1.43) and similar to the excess mRNA production in the PM range [30]. Larger FM alleles can produce mRNA as well, though to a lesser degree. For example, sample P06-32 had an unmethylated 500 CGG repeat plus a methylated 860 repeat and still produced some mRNA (0.37 ± 0.046 relative to control mean). 

Additionally, in the absence of detected unmethylated alleles, some FM alleles are still capable of producing detectable mRNA, 0- to 0.30-fold relative to the control mean (Figure 4). Sample P08-19 had large, methylated alleles that were 760 and 870 CGG repeats in size, yet produced 0.08 ± 0.0046 relative mRNA. However, it is not known whether this residual mRNA is produced by a small number of undetected *FMR1* alleles or whether methylated alleles are capable of producing low levels of mRNA. 

### 3.5. Large FM Alleles Produce Little to No FMRP, Rarely Approaching Control Levels despite Excess mRNA

**Figure 5:**
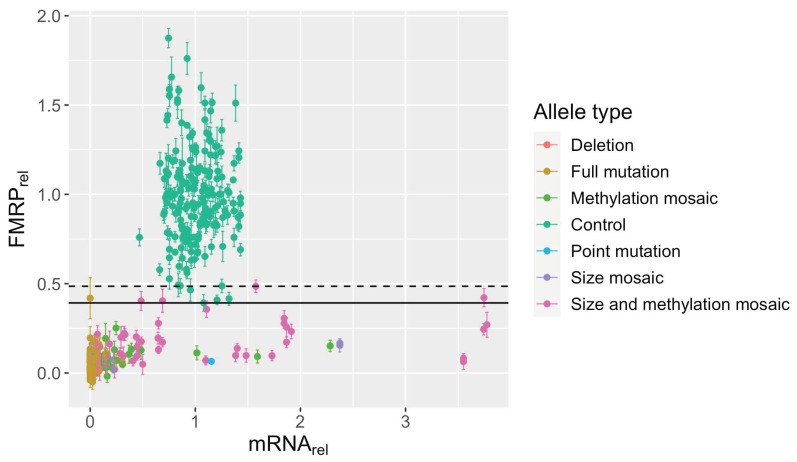
FMRP levels by mRNA level. Relative *FMR1* mRNA was plotted against relative FMRP (mean ± SE) for all allele types without controlling for subject age, CGG size, or CGG methylation status. Relative levels are normalized to that of the mean for control samples. All samples from each subject were included. Relative FMRP levels for samples with extended CGG repeats rarely approach those of control samples, regardless of mRNA level. Dashed line: maximum FMRP level of non-control samples. Solid line: minimum FMRP level of control samples.

Despite the ability of FM alleles to produce *FMR1* mRNA, they produce little to no detectable levels of FMRP. That is, samples with any form of FM allele have low levels of FMRP, thus forming a distinct group from samples with control alleles (Figure 5). However, rarely, size- and methylation-mosaic samples approached the lower bound of FMRP levels produced from control alleles; again, these observations could be due to multiple low-abundance alleles that extend into the PM range or that remain unmethylated in the low-FM range. These cases create a small range of overlap between the highest FMRP levels for size and methylation mosaics (max = 0.49) and the lower bound of control samples (min = 0.39). Notably, excess mRNA did not guarantee higher levels of FMRP, indicating the importance of the CGG repeat allele size, likely reflecting the translation efficiency, in ultimately determining the FMRP expression levels. 

FRET ratios in only 27 of 199 (~14%) non-control samples showed significant FMRP (Figure 3). Non-control FMRP-positive samples were exclusively size and/or methylation mosaics. Twenty were both size and methylation mosaics, many of which contained unmethylated PM alleles. Four had methylated PM alleles. Only three samples with purely FM alleles had evidence of FMRP: P03-10, P10-04, and P12-30 (Figure 3). Notably, all three contain unmethylated FM alleles. No sample containing only methylated FM alleles produced significant FMRP. P03-10 had methylated alleles between 470 and 800 CGGs and one unmethylated allele with approximately 250 CGGs; this sample produced *FMR1* mRNA and FMRP at levels of 2.28 ± 0.085 and 0.15 ± 0.032, respectively, relative to control means. P10-04 had a methylated 220 CGG repeat and an unmethylated 200 CGG repeat. It produced 1.6 ± 0.031 *FMR1* mRNA but only 0.09 ± 0.036 FMRP compared to the control means. P12-30 had methylated alleles between 273 and 810 CGGs and one unmethylated allele at 340 CGGs. Its relative *FMR1* mRNA was 0.25 ± 0.015 and relative FMRP levels were 0.25 ± 0.037. See Supplementary Information (Section S3) for an analysis of the accuracy of the TR-FRET assay, including Figures S1–S4.

### 3.6. Significant Association between FMR1 mRNA and FMRP in Non-Control PBMCs

**Figure 6:**
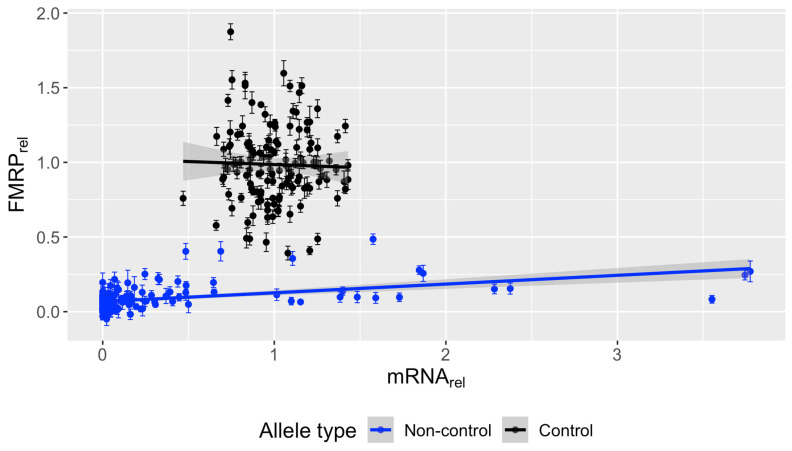
Relative FMRP positively associates with relative *FMR1* mRNA for individuals with non-control alleles. Biological and technical replicates were removed at random to generate one measurement per individual. Relative mRNA (mean ± SE) from PBMCs was plotted against relative FMRP (mean ± SE) and a linear regression was fit. No association between *FMR1* mRNA and FMRP was found for individuals with control alleles. However, *FMR1* mRNA significantly associated with FMRP in individuals with non-control alleles. Control: *n* = 134, estimate/slope = −0.041, *p*-value = 0.727. Non-control: *n* = 148, estimate/slope = 0.059, *p*-value = 1.21 × 10^−9^.

To further characterize the effect of *FMR1* mRNA on FMRP production, univariate linear regression analysis was performed using a set of representative samples, with one sample randomly chosen among biological and technical replicates per individual (separately on control and non-control samples) (Figure 6). No significant association between *FMR1* mRNA and FMRP was found in samples with control alleles (*n* = 134 unique individuals with both mRNA and FMRP values; slope = −0.041, *p* = 0.73). However, there was a significant association between relative *FMR1* mRNA and FMRP in non-control samples (*n* = 148 unique individuals with both mRNA and FMRP values; slope = 0.059 relative FMRP units per relative mRNA unit, *p* = 1.21 × 10^−9^).

**Figure 7:**
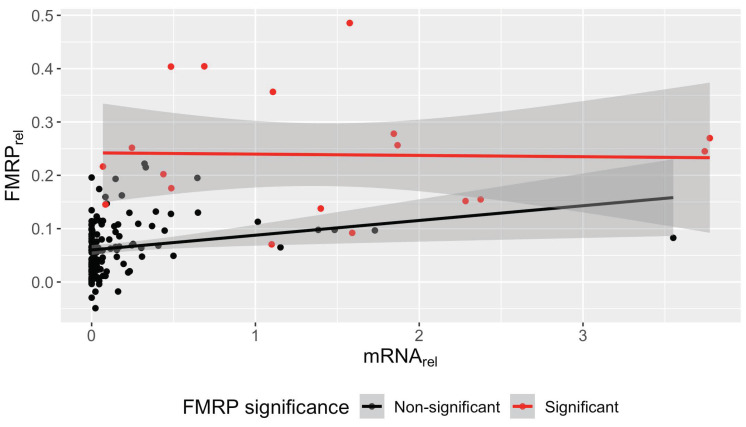
The positive association between relative *FMR1* mRNA and relative FMRP in non-control samples is driven by those with non-significant FMRP. Biological and technical replicates were removed at random to generate one measurement per subject. Relative mRNA (mean ± SE) from PBMCs was plotted against relative FMRP (mean ± SE) for non-control samples. A linear regression was fit for FMRP(+) (significant) and FMRP(−) (non-significant) samples, respectively. No association between *FMR1* mRNA and FMRP was found for FMRP(+) individuals. However, *FMR1* mRNA was significantly associated with FMRP in FMRP(−) individuals, suggesting the presence of FMRP below the level of detection via one-sample *t*-tests. FMRP(−): *n* = 131, estimate/slope = 0.028, *p*-value = 0.011. FMRP(+): *n* = 18, estimate/slope = −0.0024, *p*-value = 0.93.

The association between relative *FMR1* mRNA and FMRP in non-control samples was driven by those with the lowest FMRP values. Loess regression on mRNA versus FMRP in all non-control samples with available mRNA data (*n* = 191) shows that between 0 and ~0.5 relative mRNA, increasing mRNA leads to increasing protein. However, above ~0.5 relative mRNA, relative FMRP levels plateau at ~0.25, at which point further increases in *FMR1* mRNA no longer influence FMRP levels (Figure S5); however, FMRP levels will always depend on the RNA’s expanded CGG repeat length. Non-control samples were further analyzed separately by FMRP significance (Figure 7). FMRP(+) samples showed no significant association between *FMR1* mRNA and FMRP (*n* = 18, slope = −0.0024, *p* = 0.93). However, levels of FMRP(−) samples were positively associated with *FMR1* mRNA levels (*n* = 131, slope = 0.028, *p* = 0.011), suggesting that samples with non-significant FMRP (upon FRET analysis) do produce some FMRP, albeit at levels too low for significance testing on the basis of individual samples.

### 3.7. Translation Efficiency Is Negatively Associated with the Smallest CGG Repeat Size in Samples with FM Alleles

**Figure 8:**
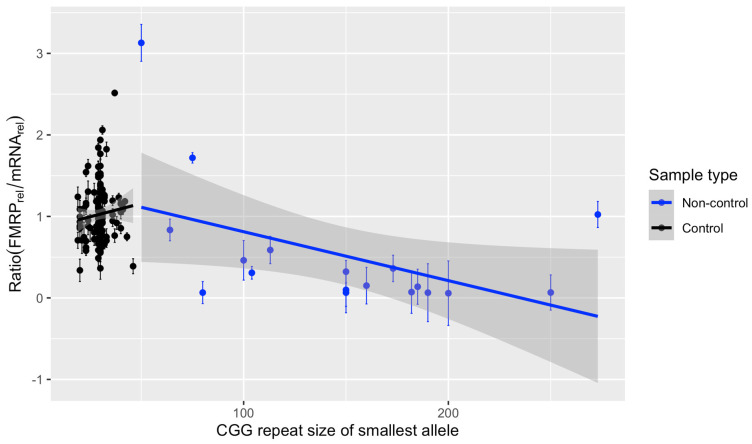
Translation efficiency by smallest CGG repeat size for samples with significant FMRP. Biological and technical replicates were removed at random to generate one measurement per individual with significant levels of FMRP. The ratio of relative FMRP to relative mRNA was used as a measure of translation efficiency and plotted against the smallest CGG repeat of a sample, regardless of methylation status. A linear regression was fit separately for control and non-control samples. Translation efficiency was independent of CGG repeat size for control alleles but showed a significantly negative correlation with repeat size for samples with non-control alleles. Control: *n* = 134, estimate/slope = 0.0065 relative efficiency units per CGG, *p*-value = 0.31. Non-control: *n* = 18, estimate/slope = −0.0060 relative efficiency units per CGG, *p*-value = 0.045.

Considering the ability of FM alleles to produce mRNA, but little protein, we next examined the ratio of relative FMRP to relative *FMR1* mRNA to approximate the efficiency of protein production in FMRP(+) individuals. The ratio of relative FMRP to mRNA was plotted against the smallest CGG repeat size regardless of methylation status (Figure 8). The smallest CGG repeat size was chosen to represent an individual’s allele most likely to contribute to protein production. A linear regression model was fitted for individuals with either control or non-control (expanded CGG repeat) alleles, respectively. No association was detected for samples with control alleles (*n* = 134 unique individuals with both FMRP and mRNA data, slope = 0.0065 relative efficiency units per CGG repeat, *p*-value = 0.31). However, a significant negative association was observed for non-control samples (*n* = 18 unique FMRP(+) individuals, slope = −0.006 relative efficiency units per CGG repeat, *p*-value = 0.045). That is, as the minimum CGG repeat size increased, less FMRP was detected for the same quantity of mRNA, suggesting a decrease in translation efficiency for alleles with larger repeats, in agreement with earlier studies [29,31].

### 3.8. No Significant FMRP Production Was Detected for Alleles Greater Than ~270 CGG Repeats

**Figure 9:**
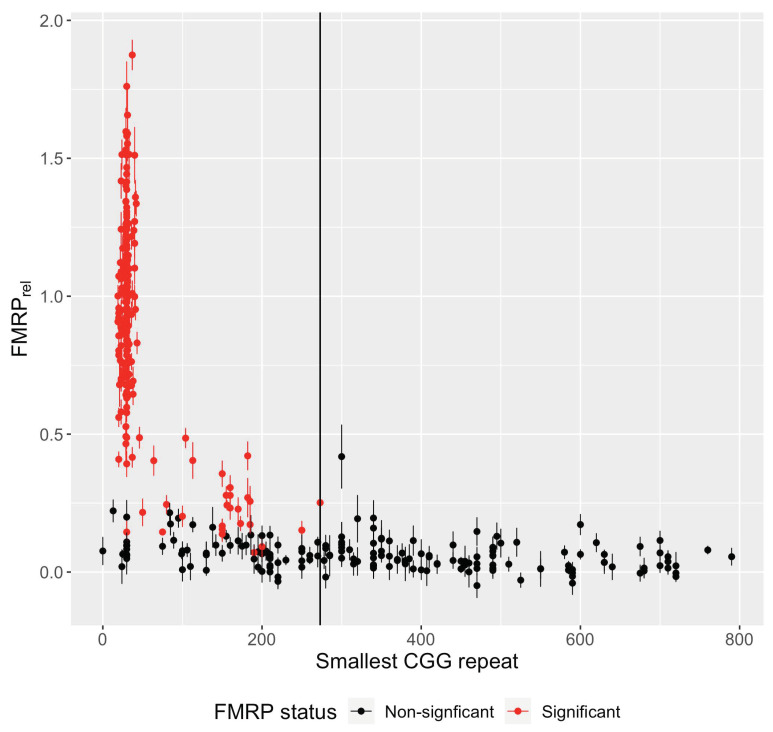
Relative FMRP by smallest CGG repeat size. Relative FMRP (mean ± SE) was plotted against the CGG repeat size of the smallest allele of each sample, regardless of methylation status. Samples were color-coded by FMRP status. No sample with a minimum allele size above 273 CGGs (solid black line) produced significant FMRP.

To estimate the largest allele capable of producing FMRP in vivo in the current study, each sample was represented by its smallest CGG repeat size regardless of methylation status and plotted against relative FMRP (Figure 9). The smallest CGG repeat size was chosen to represent an individual’s allele most likely to contribute to protein production. Sample P12-30 had the largest value for the lower bound CGG repeat, at 273 CGGs, among cells producing significant FMRP. That is, no sample with its smallest allele larger than 273 CGG repeats (from sample P12-30) produced significant levels of protein. Notably, the 273 CGG repeat allele of P12-30 was methylated. P12-30 also possessed an unmethylated 340 CGG repeat. It is unclear from these data which allele or combination of alleles produced the *FMR1* mRNA and subsequent FMRP.

### 3.9. FMR1 mRNA Significantly Affects FMRP and Unmethylated CGG Repeat Size Trends toward Significance in Nested Mixed-Effects Models

Three nested mixed-effects models were fitted to examine the effects of *FMR1* mRNA level, median unmethylated CGG repeat size, median methylated CGG repeat size, and fraction of methylation to evaluate their respective effects while accounting for the other parameters and age (see Section 2). All three models showed a significant effect of *FMR1* mRNA level on FMRP for non-control samples with median allele sizes in the FM range (*p* < 0.02 for all; Table S2, Figure S6). An LRT comparing the models with and without the unmethylated CGG repeat size (Model 2 vs. Model 3) suggested that the size of the median unmethylated CGG repeat significantly affects FMRP levels when accounting for the median methylated CGG repeat size, fraction of methylated alleles, participant’s age, and *FMR1* mRNA level (*p* = 0.0368). An LRT comparing Models 1 and 3 with and without the methylated CGG repeat and the fraction of methylated alleles suggested that neither had significant impact on FMRP levels when accounting for the unmethylated CGG repeat, participant’s age, and mRNA level (*p* = 0.6079). This finding is likely because total mRNA level encompasses the effect of the fraction of methylated (and thus unmethylated) alleles. Moreover, methylated CGGs likely contribute little to protein production, as evidenced by the fact that no sample containing only methylated FM alleles had significant levels of FMRP (Figure 3). Therefore, LRTs suggested that when *FMR1* mRNA and the unmethylated CGG repeat allele size are already accounted for, the methylated CGG repeat and the fraction of methylated alleles are no longer influential factors that could significantly contribute to the variation in FMRP production, controlling for age.

Although Model 1 showed that the median unmethylated CGG repeat size is not independently a significant contributor to FMRP (*p* = 0.0596) after accounting for *FMR1* mRNA, it suggests that doubling the length of the median unmethylated CGG repeat resulted in a 0.04% (2 × (−0.0002) × 100% = −0.04%) reduction, on average, in relative FMRP in individuals with alleles in the FM range, while fixing an individual’s age and their relative *FMR1* mRNA level at average values (see effect of CGG_unmethylated_ in Model 1 of Table S2).

## 4. Discussion

A main finding of this study is the large degree of mosaicism observed in individuals with FXS. Mosaicism was observed in three categories: (1) the number of alleles within a mutation class, (2) the number of alleles across mutation classes (size mosaicism), and (3) methylation status of each allele (methylation mosaicism). Size and methylation mosaicism for samples with FM alleles from various tissues, especially in individuals variably affected by FXS, have been observed in many studies [14,22,32,33,34,35,36,37]. In 2022, Meng and colleagues [38] also observed mosaicism in blood samples, but to a lesser degree. In the current study, only 17% of FXS cases possessed a single, fully methylated allele. All other individuals with non-control alleles had a combination of methylation or size mosaicism, including multiple FM alleles. Considering the stricter definition of size mosaicism as crossing allele class boundaries, 60% of research subjects in this study showed no size mosaicism, which closely mirrors the 69% of FXS subjects that lacked mosaicism in blood in Meng et al. [38]. Moreover, both Meng et al. [38] and the current study found similar degrees of methylation mosaicism, at approximately 12%. In contrast, Budimirovic et al. [14] found methylation mosaicism in more than half of their blood and buccal samples. Individuals with size mosaicism alone represented a smaller proportion of this study (<1%) compared to Meng et al. [38] (11%). 

Size mosaicism is likely due to the instability of the repeat tract outside of the control allele range in both meiotically and mitotically dividing cells [39,40]. The mechanisms for repeat instability at the *FMR1* locus are not well understood, but likely result from secondary structures of expanded repeats that disrupt DNA replication, repair, and/or recombination [41]. While expansions and, to a lesser degree, contractions, occur in any allele class, PM alleles are particularly meiotically unstable, often expanding into FM alleles in a single generation [40,42]. More recently, it has been shown that somatic mosaicism is also common in PM alleles and that genes involved in DNA repair may play a role in somatic expansion risk, as reported in other repeat-expansion disorders [43].

How mosaicism, both in size and methylation, affects *FMR1* mRNA expression has been surveyed. A correlation between repeat size (especially in the PM range) and (1) percent of methylation, (2) *FMR1* mRNA levels, and (3) more clinical involvement has been found [14,44], with the level of FMRP directly related to the degree of cognitive and neurodevelopmental impairment [15,45,46]. Therefore, it is unsurprising that mosaicism in individuals with FM alleles is also associated with increased expression and improved cognitive function [14,15,38,47,48]. Indeed, there is increasing evidence that unmethylated FM alleles are actively transcribed [30,36]. However, more details are needed about which specific alleles can express *FMR1* mRNA and FMRP and to what degree.

The main finding of the current study was that only samples with size and/or methylation mosaicism produced significant levels of FMRP (Figure 3). Indeed, some methylation mosaics produced excess *FMR1* mRNA, ~1.6-fold higher than the highest-producing control (Figure 4). This finding is consistent with observations of increased *FMR1* mRNA levels, sometimes 5- to 10-fold, in carriers of PM alleles [29,31] and with a case study of an unmethylated FM allele producing 7-fold excess *FMR1* mRNA [49]. 

Furthermore, FMRP was detectable in many FM-only samples but did not achieve statistical significance. Despite this, we found that in the absence of evident unmethylated alleles, some (apparently) fully methylated FM alleles are still capable of producing up to 0.30-fold detectable *FMR1* mRNA relative to the control mean (Figure 4). For example, sample P08-19 had large, methylated alleles of sizes 760 and 870 CGG repeats, yet produced 0.06 ± 0.0046 relative mRNA. Conceivably, unmethylated alleles present below the level of detection could contribute to *FMR1* mRNA levels. Furthermore, the fraction of unmethylated alleles is determined by Southern blotting and is based on the methylation status of a single CpG site (See Section 2); however, the degree of methylation along the length of each allele is unknown. Therefore, the degree of methylation may be important to identify which alleles can be transcribed and, potentially, which isoforms thereof. 

No association between *FMR1* mRNA and FMRP in control samples was observed (Figure 6). This observation is consistent with previous slot-blot [31], Luminex immunoassay [21], and TR-FRET measurements [15], and suggests that there is a limit to FMRP production beyond which there is no further metabolic drive. However, we did find an association between *FMR1* mRNA and FMRP in samples with non-control alleles as assessed via linear regression and nested mixed-effects models in the current study (Figure 6 and Figure S6). Interestingly, LOESS regression showed that FMRP increased with increasing mRNA up to ~0.5-fold relative mRNA, after which no association between mRNA and FMRP was observed (Figure S5). Therefore, the dependence of FMRP on mRNA level in non-control samples was driven by those with the lowest FMRP levels, which generally failed significance testing (Figure 7). Likely, these samples produced small amounts of FMRP below the level of significance for an individual sample, which nevertheless uncovers an association between mRNA and FMRP when taken as whole.

Despite the capacity to produce excess *FMR1* mRNA, FMRP levels remain low in FXS samples [49] (Figure 5), which is likely due to the difficulty of translation machinery in traversing secondary structure in the *FMR1* mRNA during ribosomal scanning as CGG repeats expand [31,50]. In vitro transcribed CGG repeats have been shown to produce hairpin-like structures with both CG and GG base-pair bonding and even tetraplex structures resulting from guanine quartets between two such hairpins, which would impede translation [41,51]. Indeed, the ratio of relative FMRP to relative mRNA, a measure of translation efficiency, decreased as the size of the smallest CGG repeat allele in a sample increased in the current study (Figure 8). Others have also found decreased translation efficiency in expanded repeats starting in the intermediate and PM range [31,52]. Therefore, it is unsurprising that translation efficiency would continue to decrease in the FM range.

Still, the question as to the largest CGG repeat capable of producing FMRP remains unanswered. In the current study, no sample with a minimum allele size above 273 CGG repeats produced significant levels of FMRP (Figure 9). Similarly, Feng and colleagues [50] identified low, but detectable levels of FMRP for a fibroblast clone with 285 CGG repeats via Western blotting. However, examining large repeat sizes in the absence of methylation and/or size mosaicism will more directly answer this question. This apparent threshold is of great relevance to previous and ongoing efforts to reactivate the *FMR1* gene [53,54,55], since reactivation in the absence of the ability to produce FMRP would not be a productive approach to the treatment of FXS; moreover, the production of expanded CGG repeat RNA would increase the risk of developing the late-onset neurodegenerative disorder, FXTAS [19].

*Study Strengths and Limitations*

A notable strength of the current study is our use of a large sample size (138 control individuals; 155 individuals with FXS), which allowed us to examine FMRP in the FM range via linear regression and mixed-effects modeling independent of patterns in the control or PM range. For comparison, other recent studies using two-antibody detection methods for FMRP measured fewer individuals with FM alleles: *n* = 9 [22], *n* = 31 [14], *n* = 103 [32]. 

A fundamental limitation of studies of this type is the underdetermination of the complex allelotypes, particularly for FM and methylation- and size-mosaic patterns. Identifying all alleles in a sample is challenging given the presence of smears or cryptic alleles that represent a minor but contributing fraction. Compounding this challenge is the absence of direct correspondence between capillary electrophoresis (CE) peak intensities and allele abundance, which is more pronounced for larger alleles. Due to this complexity, smears have typically been ignored in previous studies, with alleles simplified to the smallest or most prevalent allele. For modeling, this study calculated the lower and upper limit of smears and represented them as a unique allele by using the first quartile of the smear range. While it is important to include smears in our analyses, how we might best represent them is still unclear. Furthermore, total *FMR1* mRNA levels were assessed in this study. The contribution and impact of different *FMR1* mRNA isoforms was not addressed.

Finally, it remains unclear how well FMRP levels in PBMCs reflect those in the brain and therefore how useful PBMC data are for understanding neurodevelopmental disorders like FXS. Few studies have examined the correlation between blood FMRP and postmortem brain tissue FMRP in the same subject, including Pretto and colleagues [44], who showed differences in methylation status in the blood versus the brain in the PM range, and Tassone and colleagues [28], who showed tissue-specific methylation differences in PM carriers even when the size of alleles remained the same. However, our previous study did demonstrate a strong correlation between FMRP levels in peripheral tissue and IQ. Accordingly, the importance of the current work lies more in commenting on the relationships between allelotype and epiallelotypes for expanded alleles and the ability of those alleles to produce FMRP.

## Figures and Tables

**Table 1:** Descriptive statistics of participants’ age, allele size, and FMRP levels. Technical replicates were randomly eliminated to generate one measurement per participant blood draw. Biological replicates were maintained in the table to account for changes in an individual’s age and allelotype at different blood draws. Subsequent analyses randomly eliminated biological and technical replicates, so a single sample represented each individual. Smears were reduced to a single value represented by the first quartile between the low and high end of a smear. This single-value representation was added as a discrete allele to all other alleles in a sample to calculate the mean, median, and standard deviation (SD) of CGG repeat size. Minimum and maximum CGG repeat size included the low and high end of a smear. * FMRP_rel_ values for the point mutation and the deletion, determined by TR-FRET, are below the level of significance. The point mutation (position c.148 G>A) [24] prevented detection of FMRP by the Cisbio TR-FRET assay and was eliminated from FMRP analyses; FMRP was present according to Western blotting. The deletion encompasses the entire *FMR1* gene [25]. Med. = Median.

Allele Class		*n*	Age (Year)					CGG Repeat Size					FMRP_rel_				
			Min	Max	Mean	Med.	SD	Min	Max	Mean	Med.	SD	Min	Max	Mean	Med.	SD
Control		152	1	80	33.9	30.5	21.4	19	46	29.7	30	4.8	0.39	1.87	0.99	0.98	0.26
Non-control		170	0	57	11.1	8.5	10.2	13	1400	542.5	505	282.4	−0.05	0.49	0.09	0.07	0.09
	Full mutation	97	1	44	10.7	9	9.2	200	1400	610	590	245.3	−0.05	0.42	0.05	0.04	0.06
	Methylation mosaicism	22	1	32	10.6	5.5	10.2	200	1270	482.9	380	253.4	−0.02	0.25	0.09	0.08	0.06
	Size mosaicism	4	9	53	29	27	18.4	170	1240	532.8	440	364.9	0.02	0.15	0.11	0.12	0.06
	Size and methylation mosaicism	45	0	57	10.5	8	10.6	13	1540	477	440	314.7	0.01	0.49	0.17	0.14	0.12
	Point mutation *	1	-	-	10	-	-	-	-	25	-	-	-	-	0.06	-	-
	Deletion *	1	-	-	6	-	-	-	-	-	-	-	-	-	0.08	-	-

## Data Availability

We have provided a table of all data used to perform the current analysis. This table is presented as Table S1.

## Supplementary Materials

The following supporting information can be downloaded at: https://www.mdpi.com/article/10.3390/genes15030356/s1, Supplementary Information: Description of additional methods for sample elimination and outlier removal and results for assessing the accuracy of the TR-FRET FMRP assay; Figure S1: Coefficient of variance by FMRP level; Figure S2: Deviation in relative FMRP among biological replicates; Figure S3: Deviation in relative FMRP among technical replicates; Figure S4: Variance among biological and technical replicates; Figure S5: Relative mRNA levels by relative FMRP levels for non-control samples; Figure S6: Nested linear mixed-effects models showing that relative FMRP is significantly dependent on relative *FMR1* mRNA for individuals with non-control alleles; Table S1: Molecular dataset; Table S2: *FMR1* mRNA statistics for nested mixed-effects modeling.
