# Supplementary material for: Variation of FMRP Expression in Peripheral Blood Mononuclear Cells from Individuals with Fragile X Syndrome
Source: Genes (Basel). 2024 Mar 13;15(3):356. doi: 10.3390/genes15030356 (PMC10969917; doi:10.3390/genes15030356)
Supplement: Supplementary file 1 [file genes-15-00356-s001.zip › Randol_Supplementary_Information.pdf]

Supplementary information

Variation of FMRP expression in peripheral blood mononuclear cells from individuals with fragile X syndrome

Jamie L. Randol [1], Kyoungmi Kim [2,3], Matthew D. Ponzini [2,3], Flora Tassone [1,2], Alexandria K. Falcon [1], Randi J. Hagerman [2,4] and Paul J. Hagerman [1,2,*]

1 Department of Biochemistry and Molecular Medicine, School of Medicine, University of California, Davis, Davis, CA, 95616 USA
2 Medical Investigation of Neurodevelopmental Disorders (MIND) Institute, UC Davis Health, Sacramento, CA 95817, USA
3 Department of Public Health Sciences, School of Medicine, University of California, Davis, Davis, CA 95616, USA
4 Department of Pediatrics, School of Medicine, University of California, Davis, Sacramento, CA 95817, USA
* Correspondence: pjhagerman@ucdavis.edu

Methods

Section S1. *Sample elimination*
Some samples were eliminated due to insufficient protein for analysis (n = 2), loading errors (n = 16), and extreme standard deviation (n = 4).

Section S2. *Extreme outlier removal*
During FMRP quantification, we noted some wells with unexpected wavelength readings or large variation in predicted protein. As a result, we tested the electronic pipette that was used for repeated dispensing and noticed sporadic anomalous ejections, despite a majority of correct ejections. Therefore, outliers were assessed at three stages during FMRP quantification. In all stages, values were considered extreme outliers and removed from analysis if they were less than or greater than 3-fold of the interquartile range (IQR) from the first (Q1) or third (Q3) quartile, respectively: outlier < Q1-3*IQR or outlier >Q3+3*IQR. First, wells from the FRET plate were removed based on their 615 nm wavelength readings. These represented technical pipetting issues in which no conjugate or double conjugate was added to a FRET well. Next, a well was removed from analysis if its corresponding FMRPrel was an extreme outlier among the eight measurements for its sample. Finally, a sample itself (all eight measurements) was removed from analysis if its FMRPrel standard deviation was an extreme outlier among the standard deviations for all 390 samples in the current study. This situation generally resulted from pipetting issues in which more or less than 10 μL total protein was added to a FRET well and for which some replicates showed FMRP within the FM range, while other replicates showed FMRP within the normal range.

Results

Section S3. *Assessing the accuracy of the FRET FMRP assay*

The accuracy of FMRP determinations were assessed by coefficient of variation (CV) [CV (%) = 100 x σ/FMRPrel; σ = standard deviation] (Figure 1). FMRPrel levels greater than or equal to 0.5 generally had CV values less than 25% and corresponded to control samples only. Samples were then separated by FMRP significance based on one-sided one-sample t-tests of corrected FRET ratios (See Methods). For samples with significant protein and FMRPrel below 0.25, CV values ranged from ~38 to 110% and corresponded to non-control samples only. Samples with non-significant protein had much larger CVs, reflecting small and/or negative interpolated values of FMRP. Given that FMRP is approximately 4-fold lower in PBMCs compared to fibroblasts, the current results were not unexpected. Kim and colleagues (2019) found that dermal fibroblasts with FMRPrel of 0.2 generally had CVs less than 20% and fibroblasts with FMRPrel below 0.1 had CVs up to 100%. That is, FMRPrel of 0.05 in fibroblasts corresponds to ~0.2 in PBMCs, and both FMRP levels can approach CVs of ~100% in their respective studies.

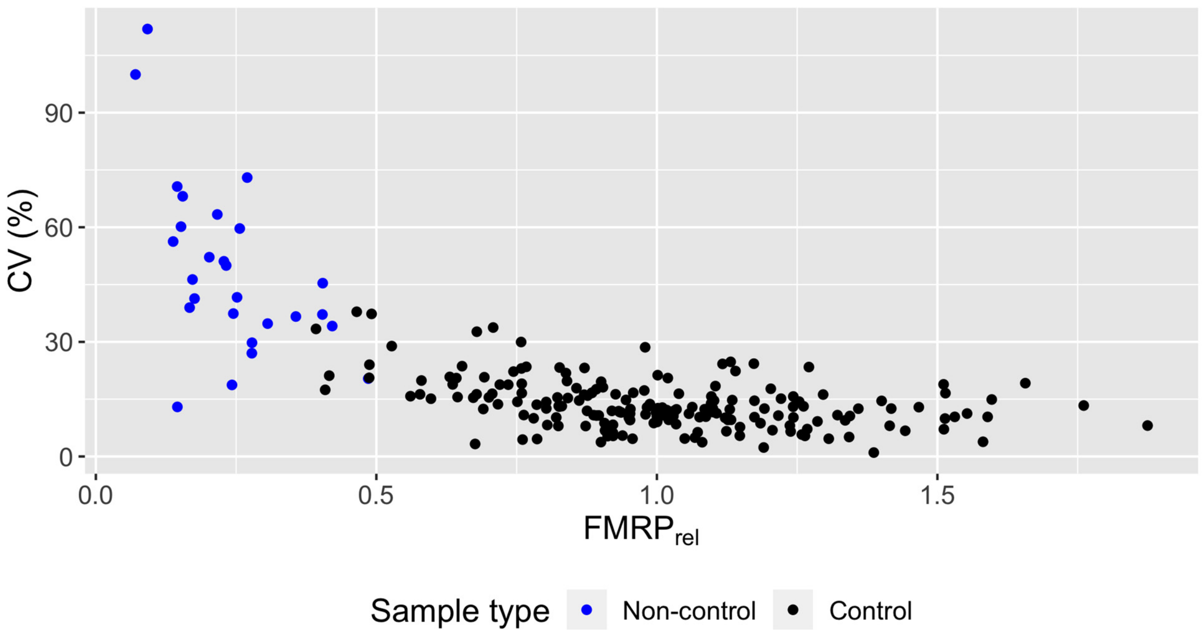

Figure S1. Coefficient of variance (CV) by FMRP level. Relative FMRP was plotted against CV (%) for all samples with significant FMRP by one-sample t-test on corrected FRET ratios (See Methods).

The FRET assay reproducibility for PBMCs was assessed by comparing FMRP significance (See Methods) and standard error of the mean (SEM) among biological replicates from the same individual measured multiple times and then among technical replicates from the same blood draw. Both were presented as a percent of their respective replicate group mean (percent variability). Of 27 individuals with multiple blood draws, only 2 (7.4%) had biological replicates that differed in significance of FMRP. That is, at least one biological replicate was FMRP(+) and at least one was FMRP(-) (Figure 2). Both individuals had non-control alleles whose corresponding relative FMRP values occurred at the transition in ability to significantly detect FMRP, indicating that the samples likely have protein, but at levels difficult to detect by significance testing. Similarly, of 68 individuals with the same blood draw run on different plates, only 4 (5.9%) had technical replicates that differed in FMRP significance (Figure 3). Again, all four samples occurred at the transition between non-significant and significant FMRP.

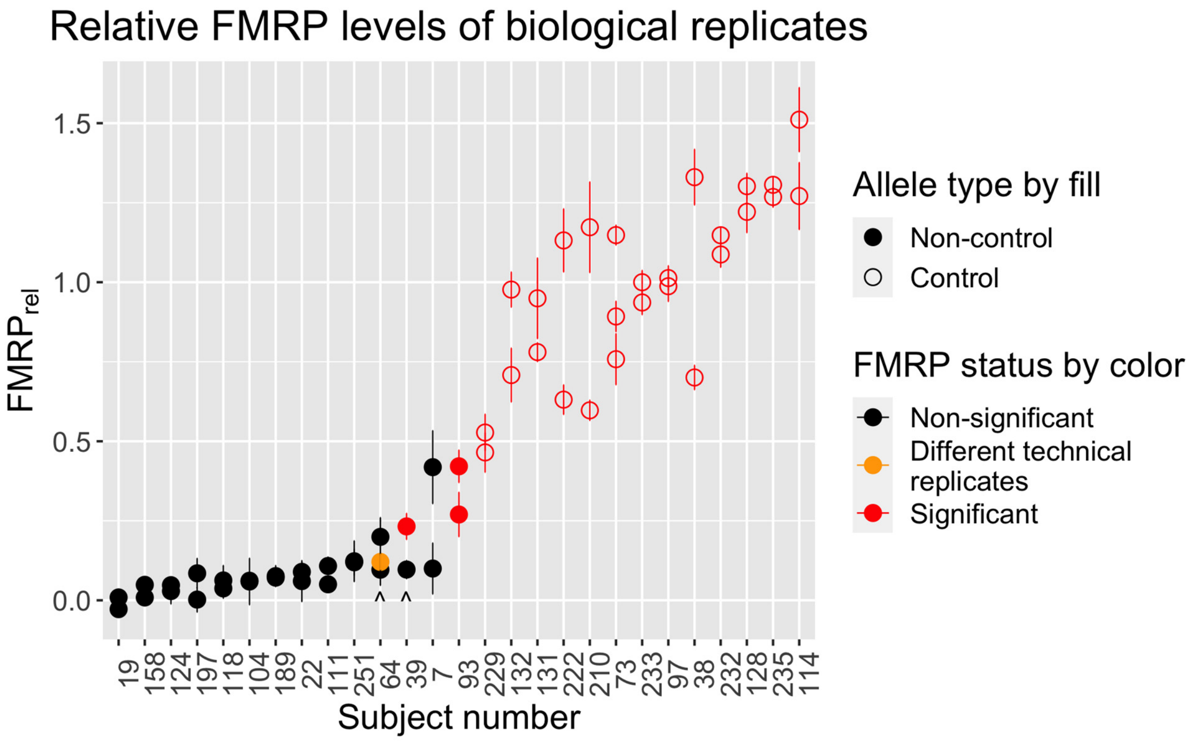

Figure S2. Deviation in relative FMRP among biological replicates. FMRP (relative to the mean of samples with control alleles) for biological replicates was plotted by an individual's unique subject number for this study (*n* = 27 individuals with biological replicates). Subject number was arranged by mean relative FMRP (mean ± SEM). Biological replicates were defined as samples from the same individual, but from separate blood draws. When biological replicates include technical replicates, the mean of the technical replicates was used. Two individuals (7.4%) with non-control alleles had relative FMRP that differed in significance between their biological replicates (^). Both occurred at the transition in ability to significantly detect FMRP, indicating that the samples likely have FMRP, but at levels difficult to detect by significance testing. "Different technical replicates" is defined as one technical replicate with significant for FMRP and the other with non-significant FMRP.

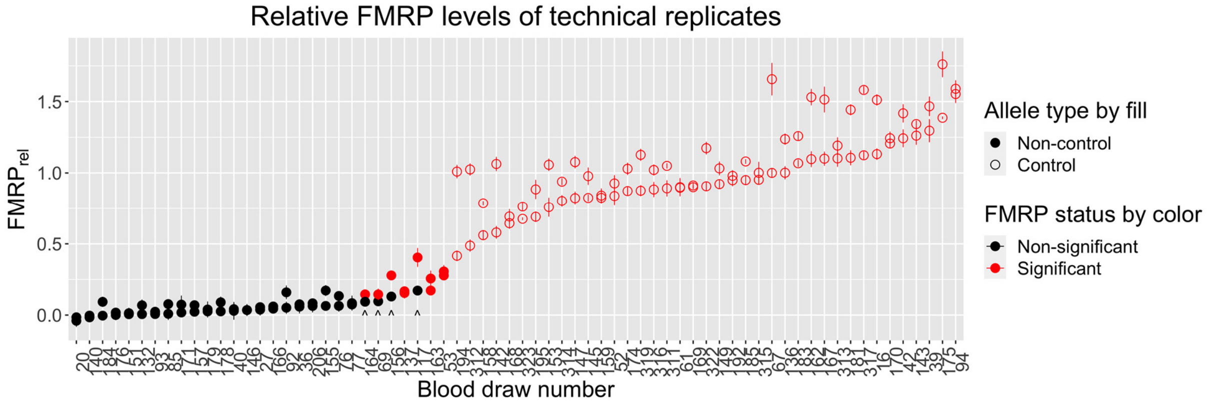

Figure S3. Deviation in relative FMRP among technical replicates. Relative FMRP for technical replicates was plotted by an individual's unique blood draw number for this study (n = 68 individuals with technical replicates). Blood draw number was arranged by minimum relative FMRP (mean ± SEM). Technical replicates were defined as samples from the same individual and the same blood draw. Four individuals (5.9%) with non-control alleles had relative FMRP that differed in significance between their technical replicates (^). All occurred at the transition in ability to significantly detect FMRP, indicating that the samples likely have FMRP, but at levels difficult to detect by significance testing.

Percent variability followed trends similar to those of CVs for all samples (Figure 1, Figure 4). For both biological and technical replicates, FMRPrel levels greater than or equal to 0.5 generally had variability values less than 25% and corresponded to control samples only. For samples with FMRPrel below 0.25, variability values ranged from -194% to 109% and corresponded to non-control samples only. High variability is consistent with small values of FMRP. A relative FMRP of 0.25 is an FMRP level that is 25% that of control PBMCs. However, given that PBMCs contain ~4-fold less FMRP than fibroblasts, 0.25 would correspond to only ~6% the level of FMRP in control fibroblasts, indicating that the assay is detecting very low levels of FMRP. Finally, no significant plate-effect was detected between technical replicates run on different plates (data not shown).

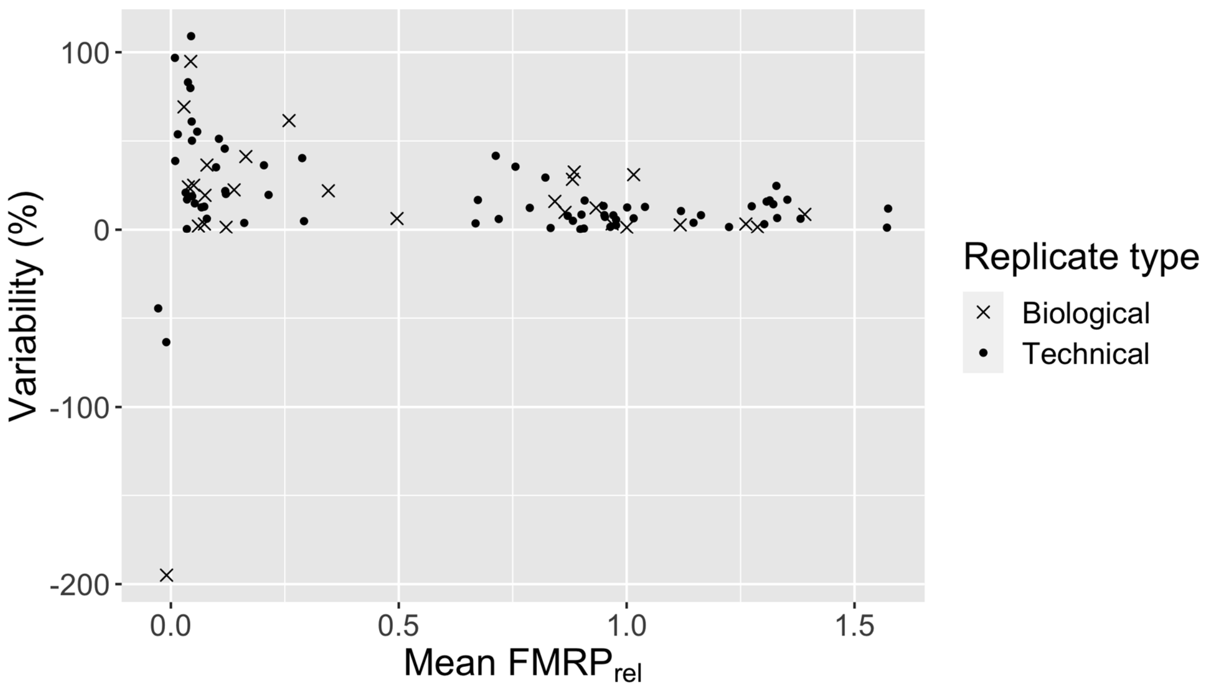

Figure S4. Variance among biological and technical replicates. Standard error of the mean (SEM) was calculated for biological and technical replicates, respectively. Variability was calculated as Variability (%)=SEM/mean × 100, and plotted against the mean relative FMRP of the replicates. SEM, rather than CV, was used to account for unequal sample size among replicate groups (n = 2-4).

Tables

Table S1. Molecular dataset.

See separate Excel file for molecular information for each sample.

Table S2. *FMR1* mRNA and median unmethylated CGG repeat statistics for nested mixed-effects modeling. Three nested mixed-effects models were generated to assess factors contributing to FMRP levels. In all three models, *FMR1* mRNA positively (positive estimate) and significantly (p-value < 0.05) contributed to FMRP levels.

Model	Function	p-value $CGG_{unmethylated}$ estimate	p-value mRNA estimate
1	$0.0565 \times mRNA + 0.0019 \times Age – 0.0002 \times CGG_{unmethylated}$	0.0596	9.3×10^{-6}
2	$0.0474 \times mRNA + 0.0027 \times Age + 0.0000289 \times CGG_{methylated} – 0.0523 \times Fraction\ Methylated$	NA	0.0090
3	$0.0420 \times mRNA + 0.0018 \times Age – 0.0002 \times CGG_{unmethylated} + 0.0000014 \times CGG_{methylated} – 0.0686 \times Fraction\ Methylated$	0.0513	0.0181
