# Supplementary figures and images for: Variation of FMRP Expression in Peripheral Blood Mononuclear Cells from Individuals with Fragile X Syndrome
Source: Genes (Basel). 2024 Mar 13;15(3):356. doi: 10.3390/genes15030356 (PMC10969917; doi:10.3390/genes15030356)

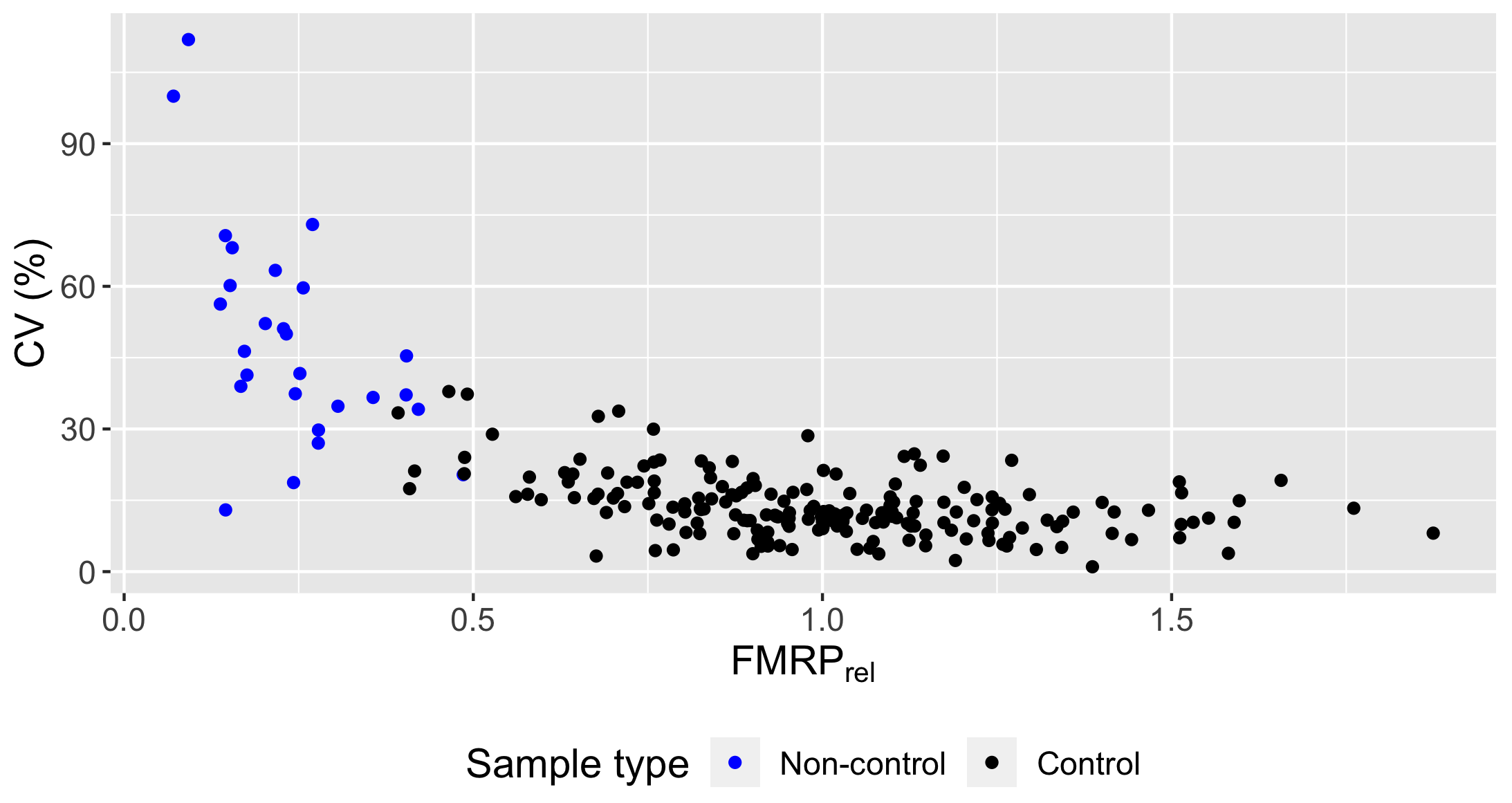

Supplement: Supplementary file 1 [file genes-15-00356-s001.zip › Figure S1.tiff]

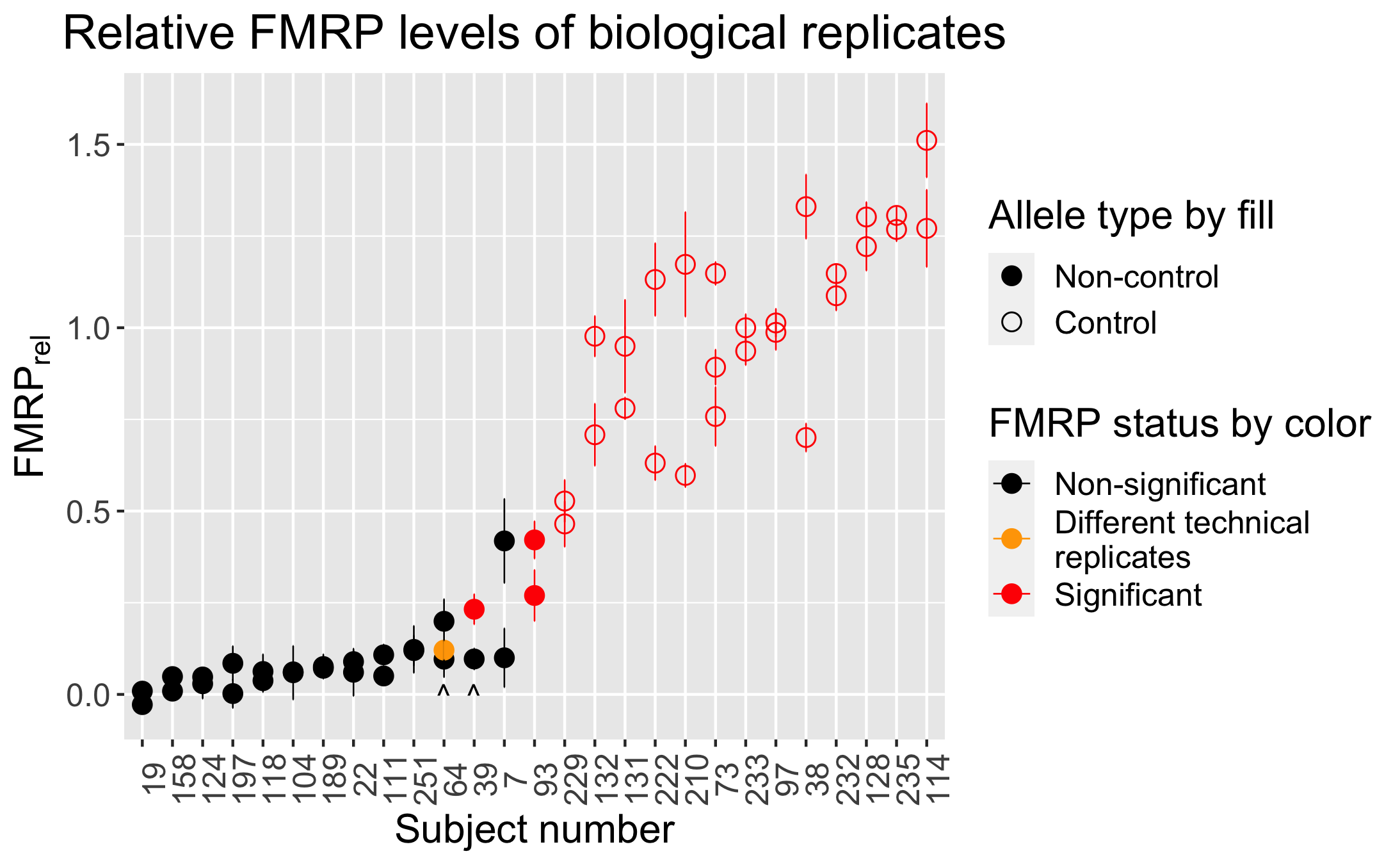

Supplement: Supplementary file 1 [file genes-15-00356-s001.zip › Figure S2.tiff]

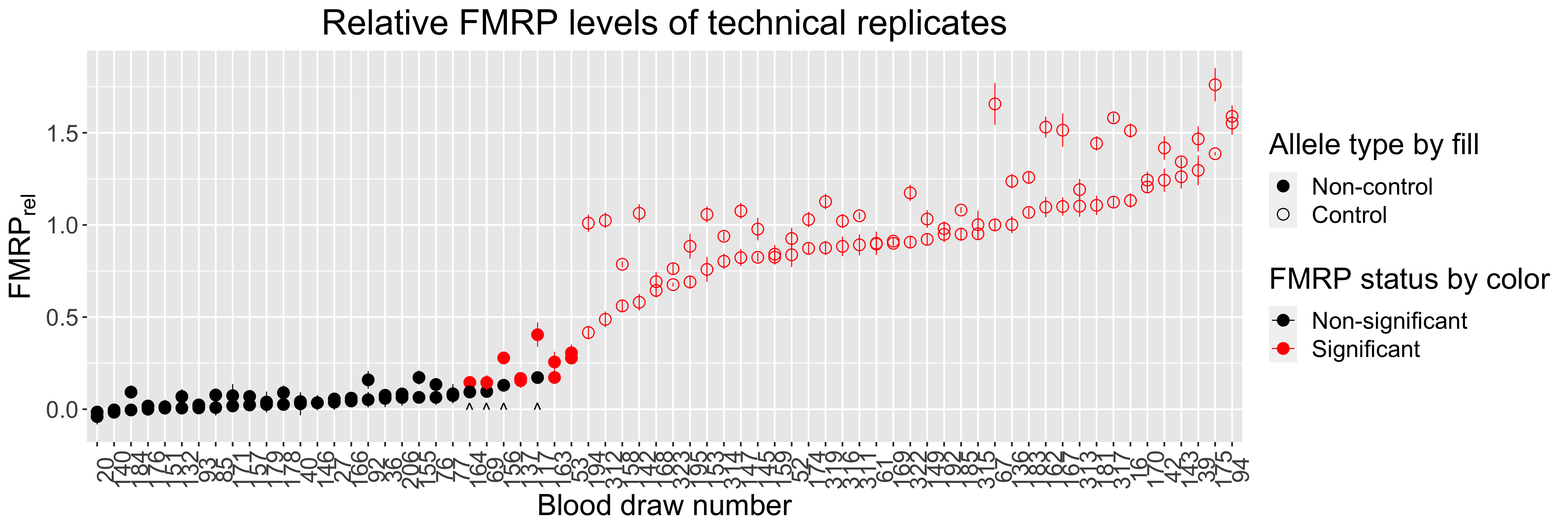

Supplement: Supplementary file 1 [file genes-15-00356-s001.zip › Figure S3.tiff]

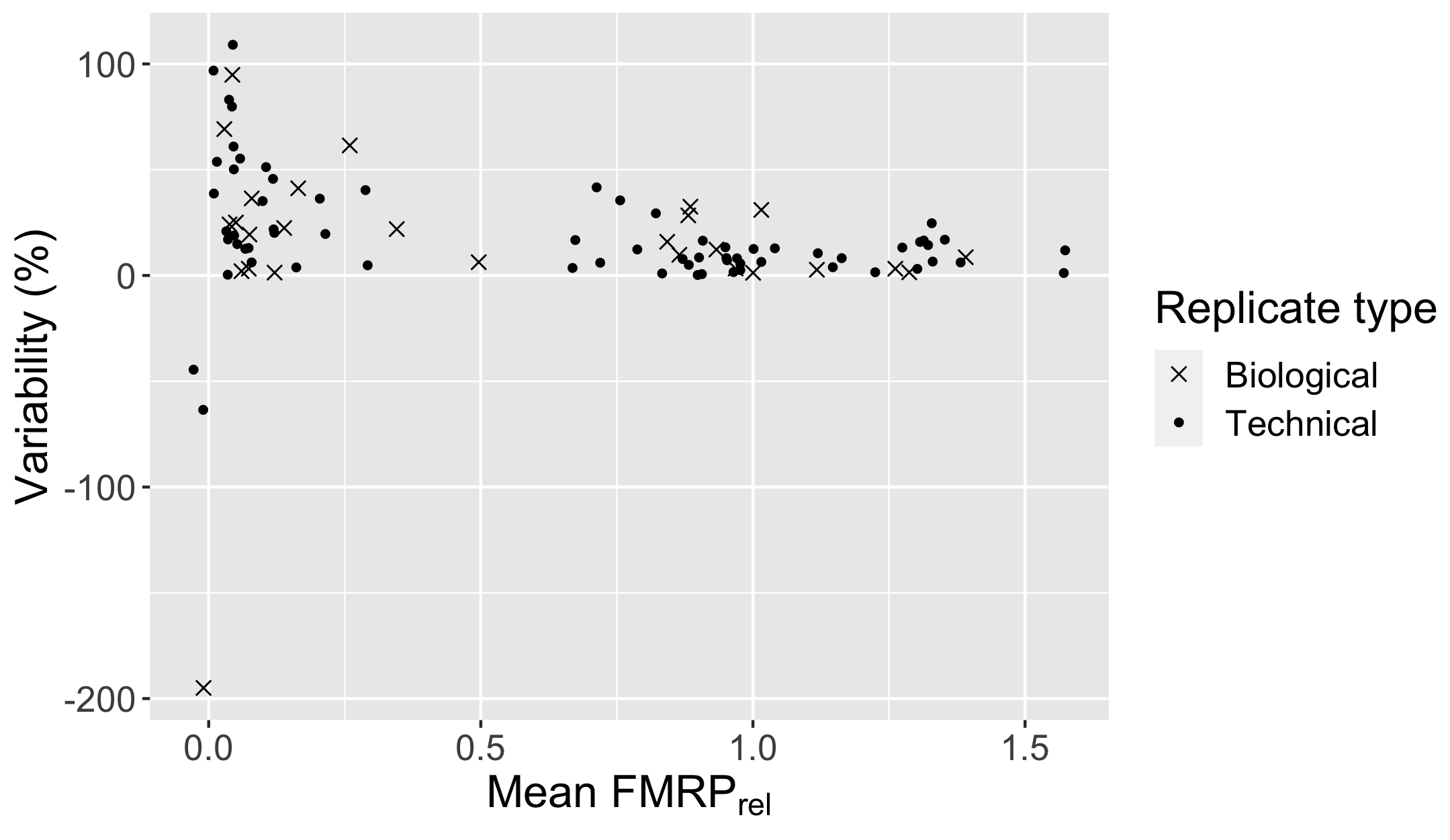

Supplement: Supplementary file 1 [file genes-15-00356-s001.zip › Figure S4.tiff]

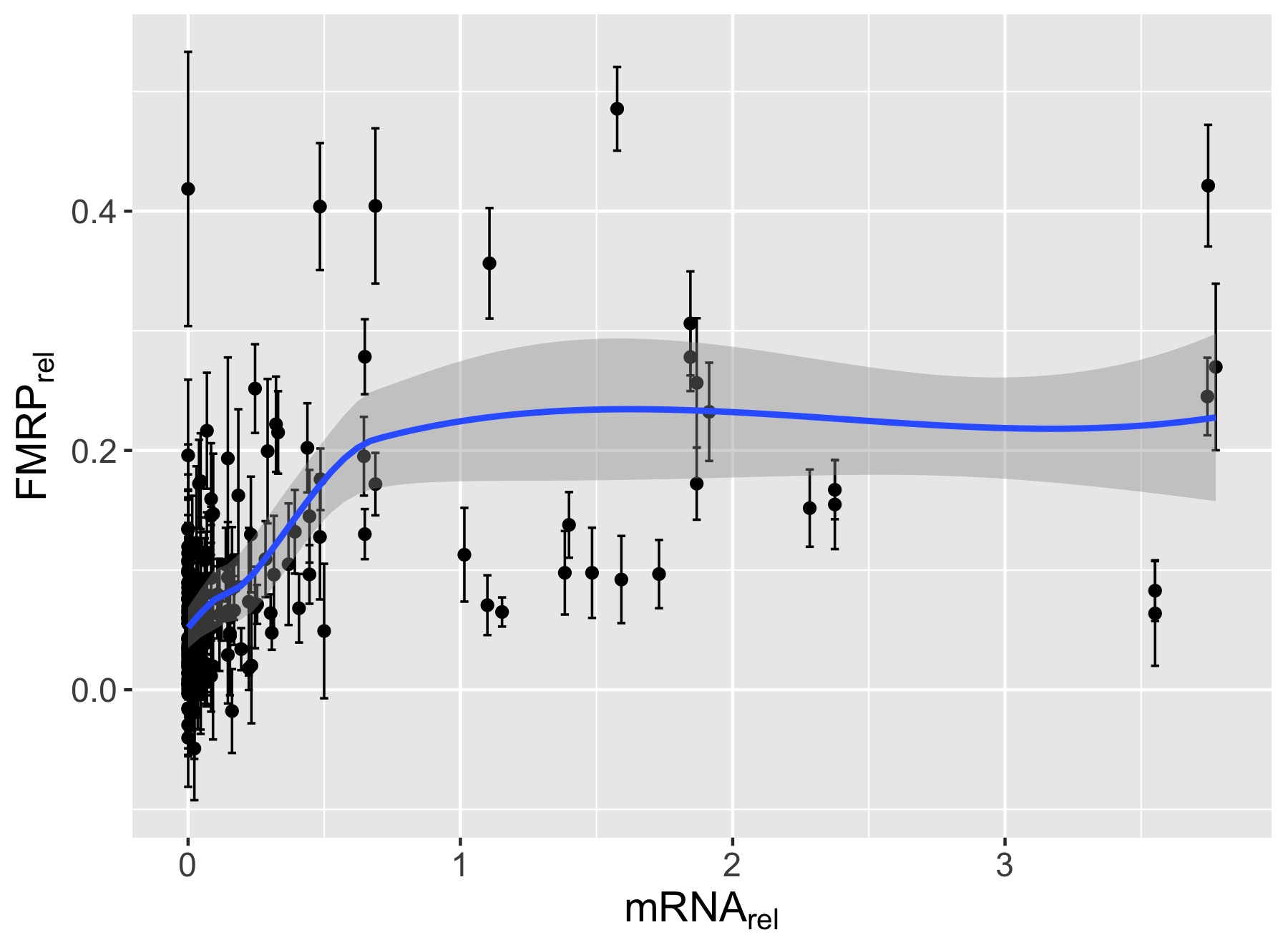

Supplement: Supplementary file 1 [file genes-15-00356-s001.zip › Figure S5.tiff]

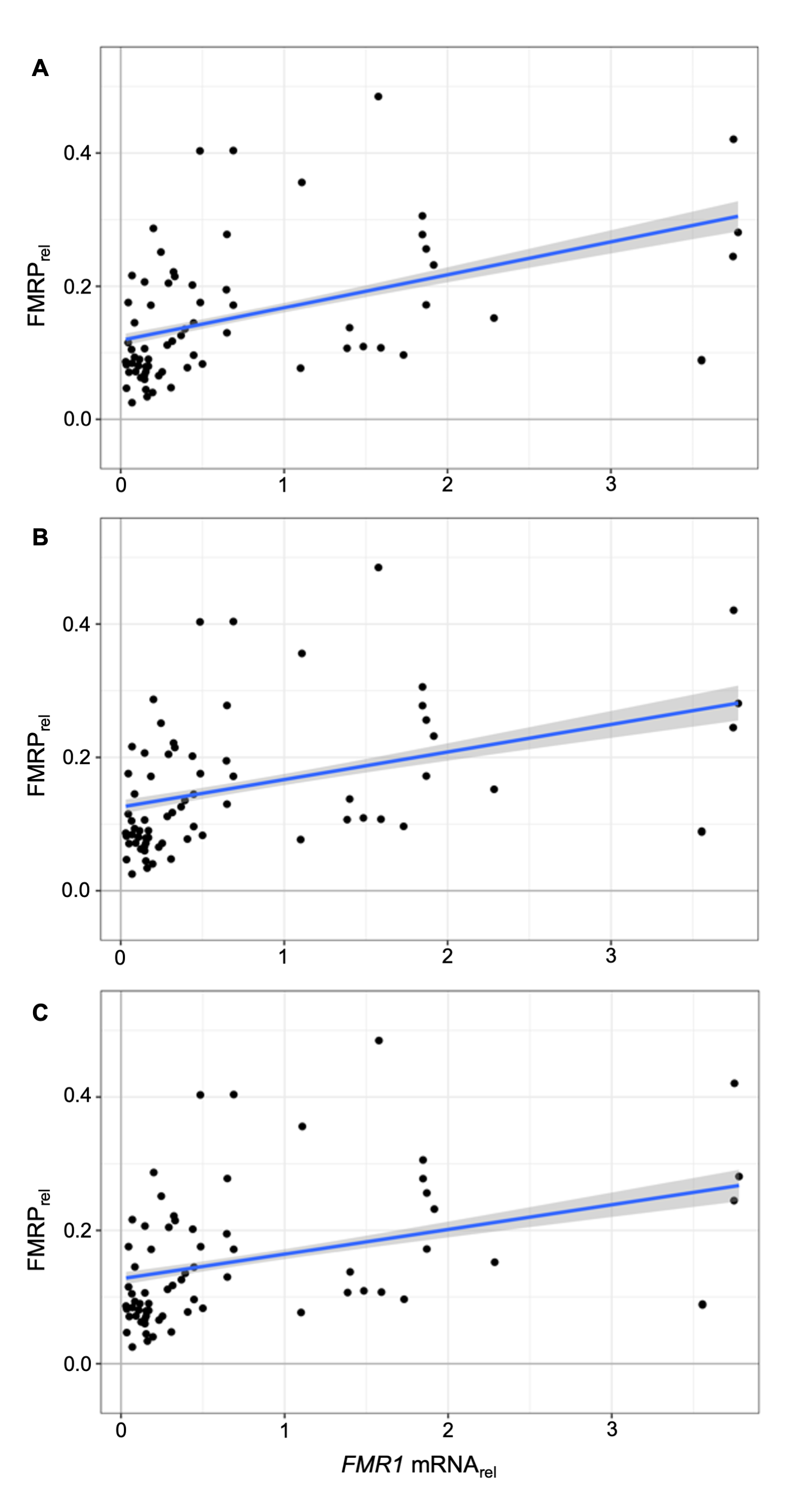

Supplement: Supplementary file 1 [file genes-15-00356-s001.zip › Figure S6.tiff]
